# Supplementary material for: Deletion of wfs1 Impairs Oligodendrocyte Precursor Cells Dorsal Distribution and Myelination Through the wfs1-hmgcs1 Axis in Zebrafish
Source: Biology (Basel). 2026 Aug 16;15(16):1402. doi: 10.3390/biology15161402 (PMC13510151; doi:10.3390/biology15161402)
Supplement: Supplementary file 1 [file biology-15-01402-s001.zip › File S1-Transcriptome sequencing data and differentially expressed genes (DEGs) in wfs1-- zebrafish at 5 dpf/2.KEGG/wfs1KOvsWT/UP/src/dre04261.html]

dre04261


- dre:100535196

- Up regulated genes

100535196(2.10467205781268)

- dre:677750

- Up regulated genes

677750(1.37913343503877)

- dre:794522

- Up regulated genes

794522(1.07749660517023)
- dre:114457

- Up regulated genes

114457\_1(1.40796876982558)

- dre:323509

- Up regulated genes

323509\_1(2.61236898874666)

- dre:568726

- Up regulated genes

568726(1.14419230813088)
- dre:560807

- Up regulated genes

560807(1.71247301071773)

- dre:556410

- Up regulated genes

556410\_1(1.7264669285964)
- dre:100538072

- Up regulated genes

100538072(1.10439883897206)

- dre:555423

- Up regulated genes

555423\_1(2.59262069341507)

- dre:559392

- Up regulated genes

559392\_1(5.40322756218621)
- dre:562282

- Up regulated genes

562282(1.6446353235091)

- dre:192322

- Up regulated genes

336121(1.42904963424916)

- dre:562552

- Up regulated genes

562552(1.50682290794657)

- dre:559392

- Up regulated genes

559392\_1(5.40322756218621)
- dre:562282

- Up regulated genes

562282(1.6446353235091)

- dre:492486

- Up regulated genes

492486(4.56703139609518)

- dre:559392

- Up regulated genes

559392\_1(5.40322756218621)
- dre:562282

- Up regulated genes

562282(1.6446353235091)

- dre:558525

- Up regulated genes

558525\_1(3.69011072176884)

- dre:793666

- Up regulated genes

793666\_1(8.27369250412896)

Close
